# Dual Effects of Presynaptic Membrane Mimetics on *α*-Synuclein Amyloid Aggregation

**DOI:** 10.3389/fcell.2022.707417

**Published:** 2022-06-07

**Authors:** Yuxi Lin, Dai Ito, Je Min Yoo, Mi Hee Lim, Wookyung Yu, Yasushi Kawata, Young-Ho Lee

**Affiliations:** ^1^ Research Center for Bioconvergence Analysis, Korea Basic Science Institute, Ochang, South Korea; ^2^ Institute for Protein Research, Osaka University, Suita, Japan; ^3^ Department of Brain and Cognitive Science, Daegu Gyeongbuk Institute of Science and Technology, Daegu, South Korea; ^4^ Biographene, Los Angeles, CA, United States; ^5^ Department of Chemistry, Korea Advanced Institute of Science and Technology, Daejeon, South Korea; ^6^ Core Protein Resources Center, Daegu Gyeongbuk Institute of Science and Technology, Daegu, South Korea; ^7^ Department of Chemistry and Biotechnology, Graduate School of Engineering, Tottori University, Tottori, Japan; ^8^ Bio-Analytical Science, University of Science and Technology, Daejeon, South Korea; ^9^ Graduate School of Analytical Science and Technology, Chungnam National University, Daejeon, South Korea; ^10^ Research Headquarters, Korea Brain Research Institute, Daegu, South Korea

**Keywords:** amyloid fibril, α-Synuclein, electrostatic interaction, helical structure, intermolecular interaction, membrane mimetic, Parkinson’s disease, presynaptic vesicle

## Abstract

Aggregation of intrinsically disordered *α*-synuclein (αSN) under various conditions is closely related to synucleinopathies. Although various biological membranes have shown to alter the structure and aggregation propensity of αSN, a thorough understanding of the molecular and mechanical mechanism of amyloidogenesis in membranes remains unanswered. Herein, we examined the structural changes, binding properties, and amyloidogenicity of three variations of αSN mutants under two types of liposomes, 1,2-Dioleoyl-sn-glycero-3-Phosphocholine (DOPC) and presynaptic vesicle mimetic (Mimic) membranes. While neutrally charged DOPC membranes elicited marginal changes in the structure and amyloid fibrillation of αSNs, negatively charged Mimic membranes induced dramatic helical folding and biphasic amyloid generation. At low concentration of Mimic membranes, the amyloid fibrillation of αSNs was promoted in a dose-dependent manner. However, further increases in the concentration constrained the fibrillation process. These results suggest the dual effect of Mimic membranes on regulating the amyloidogenesis of αSN, which is rationalized by the amyloidogenic structure of αSN and condensation-dilution of local αSN concentration. Finally, we propose physicochemical properties of αSN and membrane surfaces, and their propensity to drive electrostatic interactions as decisive factors of amyloidogenesis.

## Introduction

*α*-Synuclein (αSN), an intrinsically disordered protein consisting of 140 amino acids is abundantly expressed in the brain. Although the exact function of αSN remains unclear, recent studies suggest that it plays an important role in modulating the neurotransmitter release (Abeliovich et al., 2000; Liu et al., 2004; Burre, 2015) and protecting nerve terminals (Chandra et al., 2005). However, when exposed to stress conditions such as high levels of reactive oxygen species, soluble αSN monomers aggregate into insoluble amyloid fibrils with highly-ordered cross-*β* structures (Hashimoto et al., 1999; Souza et al., 2000; Scudamore and Ciossek, 2018). Other forms of aggregates including oligomers are also observed as an intermediate in the process of amyloid fibrillation or as a dead-end product. The abnormal *in vivo* accumulation of αSN is the pathological hallmark of synucleinopathies including Parkinson’s disease (PD), dementia with Lewy bodies, and multiple system atrophy (MSA).

The self-assembly of αSN into amyloid fibrils is characterized by two sequential steps: slow nucleation followed by rapid elongation. It is generally accepted that physicochemical and biological factors exert significant impacts on the aggregation kinetics and pathways of αSN. Namely, previous studies indicate that lagged amyloid fibril formation under physiological conditions can be accelerated by increasing temperature to 57°C or decreasing pH to 2.0 (Uversky et al., 2001). The presence of preformed amyloid seeds of lysozyme and insulin also promotes amyloidogenesis of αSN (Yagi et al., 2005). On the other hand, graphene quantum dots (GQDs), a promising carbon-based nanomaterial in biomedicine, prevent the aggregation of αSN monomers to amyloids (Kim et al., 2018). In addition to αSN, amyloid beta (Aβ) and tau also display context-dependent aggregation behaviors (Lin et al., 2019; Gee et al., 2020).

Despite highlighted expression patterns in presynaptic terminals, αSN is widely distributed in the intracellular environment and interacts with various subcellular components. Among them, lipid membranes have been increasingly accentuated due to their critical impact on the structure and aggregation propensity of αSN. Upon binding to lipid membranes, the amphipathic N-terminal region (NTR) (residues 1–∼60) and the hydrophobic non-amyloid *β* component (NAC) domain (residues ∼60–∼100) are able to adapt *α*-helical structures (Chandra et al., 2003; Georgieva et al., 2008; Dikiy and Eliezer, 2012). NMR studies at the atomic level proposed various phospholipid-binding models of αSN, i.e., the “single elongated helix” consisting of one long *α*-helix (residues 3–92) and the “broken helix” containing two curved *α*-helixes (residues 3–37 and 45–92) (Chandra et al., 2003; Jao et al., 2004; Georgieva et al., 2008; Jao et al., 2008; Bodner et al., 2009; Trexler and Rhoades, 2009; Wang et al., 2010). Recent evidence has highlighted that three different regions of αSN bind to lipid membranes in distinct structural and dynamical manners (Fusco et al., 2014). The N-terminal membrane-anchor region, consisting of the first 25 residues, binds to the membrane surface by adopting a stable helix. The central sensor segment, composed of residues 26–98, is of significant importance for the overall binding strength to lipid membranes. The C-terminal region, consisting of residues 99–140, weakly interacts with the membrane surface and remains largely disordered. Further investigations demonstrated that the initial 12 residues were partially inserted into the region occupied by the hydrophobic chains of the lipid bilayer (Fusco et al., 2016). In line with these results, the removal of residues 2–11 remarkably impairs the membrane affinity of αSN (Vamvaca et al., 2009). The distinct structures of αSN can be attributed to distinctive intermolecular interactions with membranes, which, in turn, dictate the amyloidogenicity of αSN. Along the same lines, the ratio of lipids to proteins (Galvagnion et al., 2015) and other properties of membranes including the charge of head groups and fluidity (Galvagnion et al., 2016; O'Leary et al., 2018) collectively influence the structure and amyloidogenesis of αSN. Moreover, our recent studies revealed that helical conformations in the initial structures of αSN in membranes is key to amyloid formation (Terakawa et al., 2018a).

Mutations in amyloid precursors are also crucial for regulating amyloidogenicity. For αSN, A53T and H50Q are the representative familial mutants associated with the early onset of PD, which manifest distinct aggregation behaviors and kinetics (Polymeropoulos et al., 1997; Appel-Cresswell et al., 2013; Flagmeier et al., 2016). Truncated forms of αSN are also observed in Lewy bodies in cells where a truncation at the C-terminal leads to accelerated amyloid formation (Li et al., 2005; Izawa et al., 2012; Sorrentino et al., 2018). Other reports investigate the function of highly acidic C-terminal regions of αSN in membrane binding and subsequent amyloid formation. Even upon binding to membranes, the C-terminal domain remains disordered by making only weak and transient contacts with membrane surfaces (Fusco et al., 2014). Interestingly, the removal of the C-terminal regions remarkably reshapes the kinetic factors of the aggregation propensity under membrane environments. Although recent advances in characterization techniques have promoted our understanding of the effects of biological membranes on the aggregation of αSN, much remains uncertain about the molecular and mechanical mechanisms of amyloidogenesis of αSN in membranes.

Herein, we investigated mainly the impacts of presynaptic vesicle-mimicking model (Mimic) membranes on the amyloid fibrillation of αSN. Collective results from the structural change, membrane binding, and amyloid fibrillation of three αSN variants demonstrated that negatively charged Mimic membranes induce biphasic modulation of the amyloidogenicity of αSN. To explain this dual effect, i.e., promotion and inhibition, we propose two mechanisms based on the amyloidogenic structure of αSN and the condensation-dilution of local αSN concentration in membranes. Taken together, this study establishes a general mechanistic perspective on the amyloid fibrillation of αSN in membranes and thereby contributes to the rational design of candidates against its deleterious aggregation.

## Materials and Methods

### Materials

The full-length human αSN (αSN_WT_) and three variations of αSN mutants: 1) C-terminal 11-residue truncation (αSN_129_); 2) charge neutralization of negatively-charged residues between positions 130 and 140 to asparagine residues (αSN_130CF_); 3) mutation of the 53rd residue from alanine to threonine (αSN_A53T_), were expressed in E. coli BL21 (DE3), and purified as previously described (Izawa et al., 2012). Phospholipids, DOPC, 1,2-Dioleoyl-*sn*-glycero-3-Phosphoethanolamine (DOPE), and 1,2-Dioleoyl-*sn*-glycero-3-Phospho-l-serine (DOPS) were obtained from Avanti Polar Lipids Inc. (Alabaster, United States) (Supplementary Figure S1). Thioflavin T (ThT) was purchased from Wako Pure Chemical Industries, Ltd., (Osaka, Japan). All other reagents were obtained from Nacalai Tesque (Kyoto, Japan).

### Vesicle Preparation

Small unilamellar vesicles (SUVs) containing DOPC or DOPC:DOPE:DOPS at a ratio of 2:5:3 were prepared as mimicking presynaptic vesicles according to the previous literature (Terakawa et al., 2018a). Briefly, lipids were dissolved in chloroform, and mixed in glass tubes at the desired compositions. The resulting solution was dried under a nitrogen stream, followed by vacuum drying to ensure the removal of residual organic solvents. To rehydrate the resultant lipid film, a solution of 20 mM sodium phosphate buffer (pH 7.4) containing 100 mM NaCl was added with vortex mixing. After 10 freeze-thaw cycles, lipid suspensions were sonicated for 10 min on ice to obtain a homogeneous SUVs solution.

### ThT Fluorescence Assay

αSNs were dissolved in 20 mM sodium phosphate buffer (pH 7.4) containing 100 mM NaCl to prepare a stock concentration of 200 μM. Protein concentrations were determined using the UV-absorbance at 280 nm with molar extinction coefficients of $2980M^{-1}\cdot cm^{-1}$ for αSN_129_, and $5960M^{-1}\cdot cm^{-1}$ for αSN_WT_, αSN_130CF_, and αSN_A53T_. The following experimental conditions were used to investigate αSNs amyloid formation at 37°C: 50 μM αSNs, 20 mM sodium phosphate buffer (pH 7.4), 100 mM NaCl, 5 μM ThT, and Mimic and DOPC model membranes at various concentrations of lipids. Sample solutions (200 μl) were applied in triplicate to each well of the 96-well microplate (Greiner-Bio-One, Tokyo, Japan), and sealed with a film (PowerSeal CRISTAl VIEW, Greiner-Bio-One, Tokyo, Japan). The microplate, placed on a water bath-type ultrasonic transmitter (Elestein SP070- PG-M, Elekon Sci. Inc., Chiba, Japan), was subjected to cycles of ultrasonication for 1 min at 9-min intervals. The fluorescence intensity of ThT was hourly recorded on an SH-9000 microplate reader (Corona Electric Co., Ibaraki, Japan) with excitation and emission wavelengths of 450 and 485 nm, respectively.

After the data acquisition, kinetic analyses of αSNs amyloid formation were carried out using the following equation:

$$Y=y_{i}+m_{i}t+\frac{y_{f}+m_{f}t}{1+\exp\left[-k\left(t-t_{0}\right)\right]}$$ (1)

where $y_{i}+m_{i}t$ and $y_{f}+m_{f}t$ are the initial and final baselines, respectively. $t_{0}$ is the half-time at which ThT fluorescence reaches 50% of the maximum amplitude. k represents the elongation rate constant. The lag time was obtained based on the following relationship: $lag\;time=t_{0}-2\left(1/k\right)$ (Nielsen et al., 2001). $y_{i}$ and $y_{f}$ were fixed to the values of initial and final ThT fluorescence intensities obtained from measurements. ThT data were fitted with the variation of $m_{i}$ , $m_{f}$ , k , and $t_{0}$ . The average and error values of the lag time and elongation rate constant were calculated from three separate samples in a single set.

### Isothermal Titration Calorimetry

Isothermal titration calorimetry (ITC) experiments for Mimic and DOPC membranes at 25°C were performed with ITC_200_ and Auto-ITC_200_ instruments (Malvern Panalytical, United Kingdom), respectively. The concentration of αSN in the ITC syringes was 400 μM. The concentration of the lipids of Mimic and DOPC membranes in the ITC cell was 2 mM. αSNs were dissolved in 20 mM sodium phosphate buffer (pH 7.4) containing 100 mM NaCl. The reference power was set to $10µcal\cdot \sec^{-1}$ , and the initial delay was 300 s. Titration experiments consisted of 20 injections spaced at intervals of 300 s. The injection volume was 0.4 µl for the first injection and 2 µl for the residual injections. The stirring speed was 1,000 rpm. Data were analyzed with a one-set of sites binding model using the MicroCal PEAQ-ITC Analysis Software (Malvern Panalytical, United Kingdom). The equation for this binding model was (Nuscher et al., 2004):

$$Q=\frac{N^{*}{\left[S\right]}_{t}\Delta H^{*}V_{0}}{2}\left[1+\frac{L_{R}}{N^{*}}+\frac{{K_{d}}^{*}}{N^{*}{\left[S\right]}_{t}}-\sqrt{{\left(1+\frac{L_{R}}{N^{*}}+\frac{{K_{d}}^{*}}{N^{*}{\left[S\right]}_{t}}\right)}^{2}-\frac{4L_{R}}{N^{*}}}\right]$$ (2)

where Q represents the change in the heat values in the system, and ${\left[S\right]}_{t}$ and $V_{0}$ denote the total concentration of the samples in the cell and the total volume of the cell, respectively. $L_{R}$ is the ratio of the total concentration of the samples in the syringe to ${\left[S\right]}_{t}$ at any given point during titration. $N^{*}$ is the binding stoichiometry of the protein per lipid molecule. The experimental data were fitted by the variation of $N^{*}$ and the molar enthalpy for binding $\Delta H^{*}$ , as well as the microscopic dissociation constant, ${K_{d}}^{*}$ . The independent lipid-binding sites per protein molecule (*N*) was calculated as $N=1/N^{*}$ . With respect to the protein, the enthalpy per mole of protein ( ΔH ) and the macroscopic dissociation constant, $K_{d}$ , were calculated based on $\Delta H=N\Delta H^{*}$ and $K_{d}={K_{d}}^{*}/N$ , respectively. The free energy per mole of protein ( ΔG ) and the entropy per mole of protein ( ΔS ) were calculated from the relationships $\Delta G=-RT\ln K_{d}$ and ΔS=(ΔH−ΔG)/N **.**

## Results

### Structural Characterization of αSN Mutants Under Membrane Environments

To characterize the size of Mimic and DOPC SUVs, we performed dynamic light scattering measurements. The hydrodynamic radius (*R*_H_) of Mimic and DOPC SUVs were estimated to be 34.2 ± 0.3 and 26.2 ± 2.1 nm, respectively (Supplementary Figure S2). These results are in line with previous reports on SUVs prepared by ultrasonication (Shvadchak et al., 2011; Kinoshita et al., 2017; Terakawa et al., 2018a).

**FIGURE 1:**
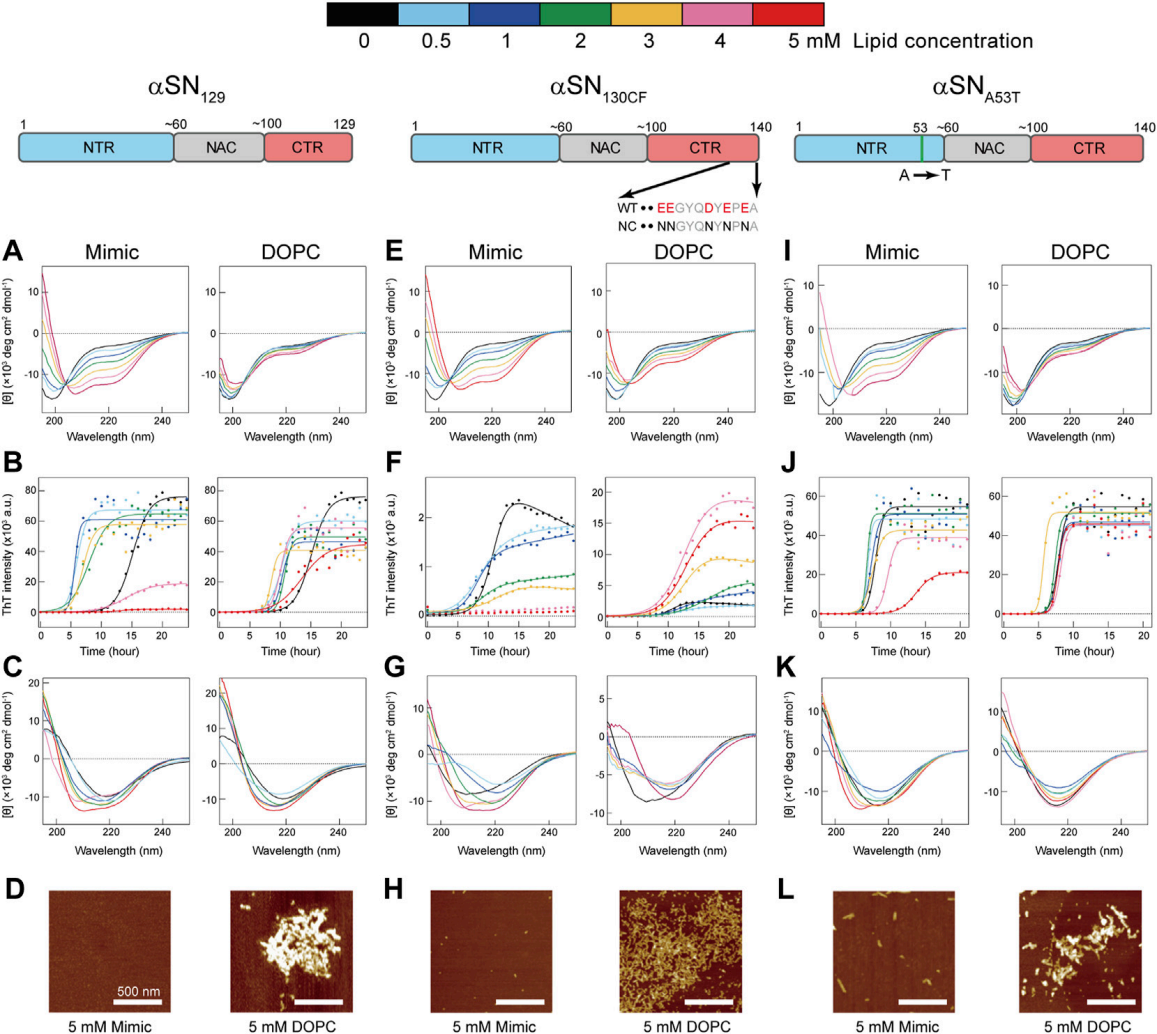
Effects of model membranes on the structure and amyloid formation of αSNs. **(A–L)** Conformational transitions and fibrillation kinetics of αSN_129_ **(A–D)**, αSN_130CF_ **(E–H)**, and αSN_A53T_ **(I–L)** in the absence and presence of Mimic (left) and DOPC membranes (right). Far-UV CD spectra of αSN_129_ **(A,C)**, αSN_130CF_ **(E,G)**, and αSN_A53T_ **(I,K)** before **(A**,**E**,**I)** and after **(C,G,K)** incubation were acquired. **(B**,**F**,**J)** Fibrillation kinetics of αSN_129_ **(B)**, αSN_130CF_ **(F)**, and αSN_A53T_ **(J)** were monitored by the ThT fluorescence assay. Raw data averaged from three separate samples are shown as closed circles. Solid lines represent the fit curves. Schematic representations of αSN_129_, αSN_130CF_, and αSN_A53T_ are displayed above the corresponding data. The *N*-terminal region (NTR), the non-amyloid *β* component (NAC) region, and the *C*-terminal region (CTR) are colored in blue, grey, and red, respectively. Various concentrations of lipids in Mimic and DOPC membranes are guided by distinct colors: black (0 mM), light blue (0.5 mM), blue (1 mM), green (2 mM), yellow (3 mM), pink (4 mM), and red (5 mM). **(D**,**H**,**L)** AFM images were taken for the samples of αSN_129_ **(D)**, αSN_130CF_ **(H)**, and αSN_A53T_ **(L)** incubated with 5 mM of Mimic (left) or DOPC (right) lipids. The white scale bars indicate 500 nm.

Far-UV circular dichroism (CD) spectroscopy elucidates the effects of Mimic and DOPC membranes on the initial structures of three different αSN variants–αSN_129_, αSN_130CF_, and αSN_A53T_ (Figures 1A,E,I). αSN_WT_ shows a unique charge cluster in its C-terminal part. The C-terminal region spanning positions 104 and 140 contains 14 amino acids that are negatively charged under physiological conditions. αSN_129_ produced with the deletion of 11 residues from the C-terminal region of αSN_WT_ reduces 5 acidic amino acid residues compared to αSN_WT_. αSN_130CF_ has an identical number of acidic amino acid residues of αSN_129_ due to the charge neutralization of acidic residues between positions 130 and 140 to asparagine residues. These two variants were mainly designed to elucidate the impact of the negative charge of the C-terminal parts on amyloid formation of membrane-bound αSN. On the other hand, αSN_A53T_, familial mutant in PD (Polymeropoulos et al., 1997), was introduced to investigate the role of a helical conformation for amyloidogenesis on membranes as Ala 53 is located in a helical structure of αSN on membrane surfaces (Chandra et al., 2003; Georgieva et al., 2008). In the absence of membranes, αSN_129_ exhibited a single negative band at ∼200 nm without any noticeable band in the region between 210 and 230 nm, indicating that the secondary structures are predominantly disordered. On the other hand, increasing the concentration of Mimic lipids from 0 to 5 mM induced helix-rich conformations as characterized by the two negative bands at ∼208 and ∼222 nm (Figure 1A, left). Further secondary structure analysis showed consistent results with increased helical structures and decreased *β*- and random-coil structures as a function of Mimic lipids concentration (Supplementary Figure S3).

In contrast to Mimic membranes, DOPC membranes caused negligible intensity magnifications in the negative peaks of CD spectra. Even after increasing the concentration of DOPC lipids to 5 mM, a minor structural alteration of αSN_129_ upon binding was still elicited (Figure 1A, right). Similar structural reconfigurations to those of αSN_129_ were also observed for αSN_130CF_ and αSN_A53T_ in the presence of Mimic and DOPC membranes (Figures 1E,I). These results indicate that Mimic membranes are more effective in generating helical structures of αSNs, which corroborate our previous findings with αSN_WT_ (Terakawa et al., 2018a).

### Calorimetry-Based Investigation of Intermolecular Interactions Between Mimic Membranes and αSNs

**FIGURE 2:**
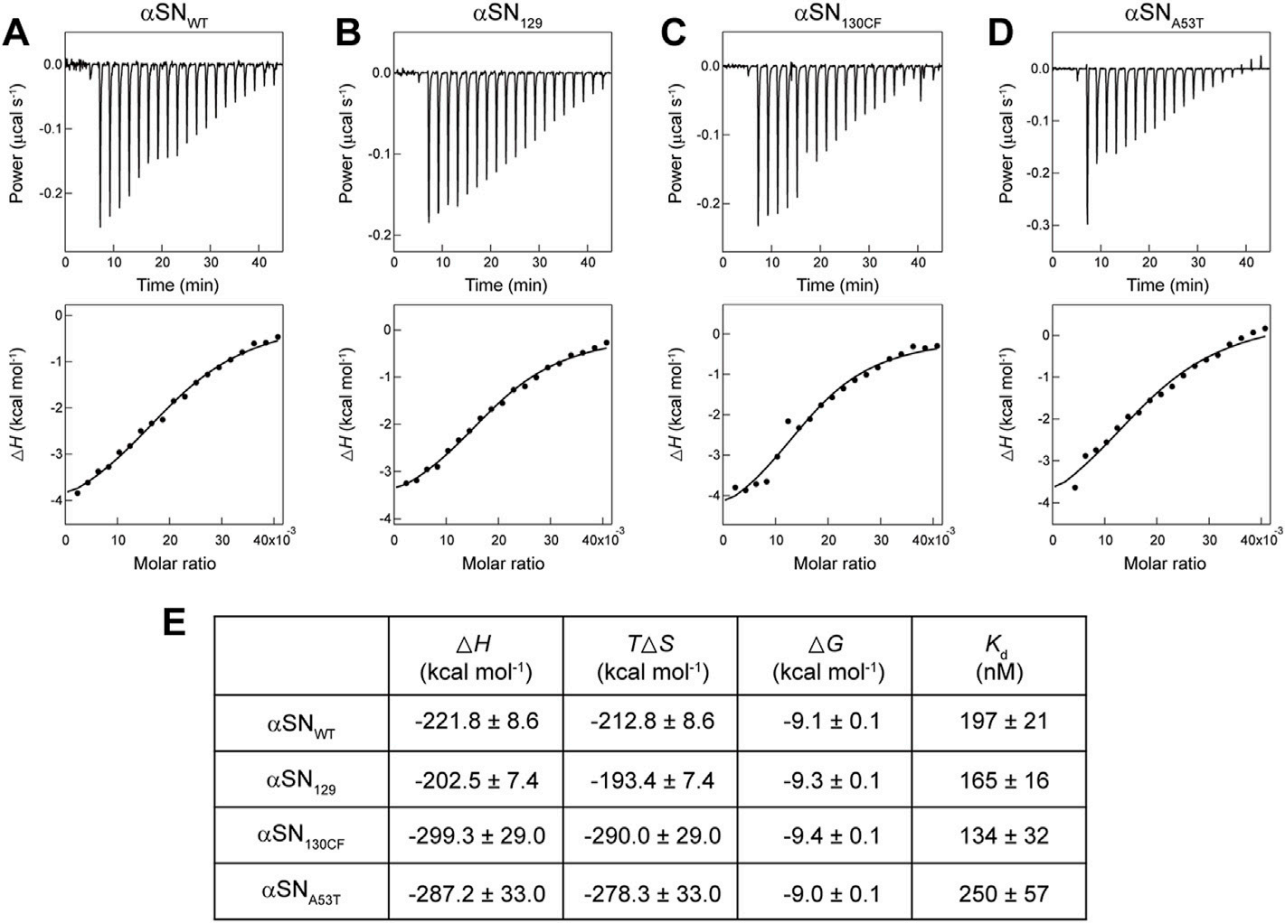
Calorimetry-based characterization of interactions between αSNs and Mimic membranes. **(A–E)** ITC thermograms (upper) and binding isotherms (lower) obtained by titrating αSN_WT_ **(A)**, αSN_129_ **(B)**, αSN_130CF_ **(C)**, and αSN_A53T_ **(D)** to Mimic membranes are shown. Solid lines in binding isotherms indicate the fit curves based on a one-set of sites binding model. **(E)** Thermodynamic parameters for the binding of αSNs to Mimic membranes.

To obtain further insights into the binding of αSN to membranes, we performed ITC analysis on αSN-membrane interactions. As shown in Figures 2A–D (upper), a series of titration of αSNs to Mimic membranes generated negative ITC peaks followed by gradual saturation. This suggests the presence of appreciable exothermal intermolecular interactions between αSNs and Mimic membranes. Following normalization of all ITC peaks, ITC thermograms were converted to binding isotherms (Figures 2A–D, lower). Although patterns of heat flow in the ITC thermogram appeared to be flat, ΔH plots in the binding isotherm were fit with a one-site binding model. The obtained thermodynamic parameters are summarized in Figure 2E. It should be noted that the binding isotherms were not the typical sigmoidal shape, which may lower the accuracy of thermodynamic parameters obtained by a fitting analysis.

As expected from downward ITC peaks, thermodynamically favorable enthalpy changes ( ΔH<0 ), ranging from $∼-200\;to\;∼-300kcal\cdot mol^{-1}$ , ensued for all variants of αSN. A large negative value of Δ*H* may stem from the membrane binding of αSNs being steered by attractive electrostatic interactions and concomitant helical folding. On one hand, unfavorable negative entropy change ( TΔS<0 ) were monitored from $∼-190\;to\;∼-290kcal\cdot mol^{-1}$ . Nevertheless, the loss of conformational and translational entropies owing to the membrane-induced helical folding of αSNs and the restricted lipid diffusion were compensated by large negative ΔH resulting in thermodynamic stabilization. The outcomes demonstrate that αSNs-Mimic membrane interactions are purely driven by the enthalpy change. Similar trends entailed for the interactions between αSN_WT_ and model membranes which consisted of phosphatidylserine and gangliosidosis-1 (Nuscher et al., 2004; Bartels et al., 2014). In contrast, no noticeable change in the ITC thermogram and binding enthalpy was observed for αSN_WT_-DOPC interactions (Supplementary Figure S4), indicating a weak interaction between αSN_WT_ and DOPC membranes. This result also suggests the key role of negatively charged lipids in the interaction with αSN.

ITC analyses provided distinct dissociation constant ( $K_{d}$ ) for all binding systems which are within a similar range. The changes in the Gibbs free energy ( ΔG ) showed negative values ranging from $-9.0\;to\;-9.4kcal\cdot mol^{-1}$ , which indicate spontaneous interactions of all αSN variants with Mimic membranes. It should be noted that the binding affinity decreased in the order of αSN_130CF_, αSN_129_, αSN_WT_, and αSN_A53T_. Altogether, the findings from the ITC study suggest that the negative charges of the C-terminal region play a pivotal role in the thermodynamic adjustment of αSN upon binding to Mimic membranes.

### Amyloid Formation of αSN Mutants Under Membrane Environments

ThT fluorescence assay examines the aggregation behaviors of the three αSN mutants with ultrasonication in the absence and presence of the test membranes. Amyloid formation of αSN under quiescent conditions is markedly slow, generally taking more than several days, with large fluctuations in aggregation kinetics (Hsu et al., 2009; Buell et al., 2014). Mechanical agitation, such as stirring and shaking, has been widely used to accelerate αSN amyloid generation *in vitro* studies (Uversky et al., 2002; Grey et al., 2011). Ultrasonication has also been introduced as an effective amyloid inducer by disrupting the metastability of supersaturation (Yoshimura et al., 2012; Lin et al., 2014; Yagi et al., 2015; Terakawa et al., 2018a). Our previous study revealed that sonication is also applicable to αSN amyloid fibrillation in membrane environments (Terakawa et al., 2018a). DLS results revealed 24-h incubation with ultrasonication did not induce an appreciable change in the size of the two types of SUVs. (Supplementary Figure S2). It should be noted that mechanical treatments such as ultrasonication may disrupt the integrity of lipid bilayers (Pandur et al., 2020), which might cause the insertion of αSNs into the lipid bilayers. Even if there might be an effect of sonication on membranes, the comparison of results of αSN_WT_ with those of variants will be still valid as they were exposed to the same environmental changes.

**FIGURE 3:**
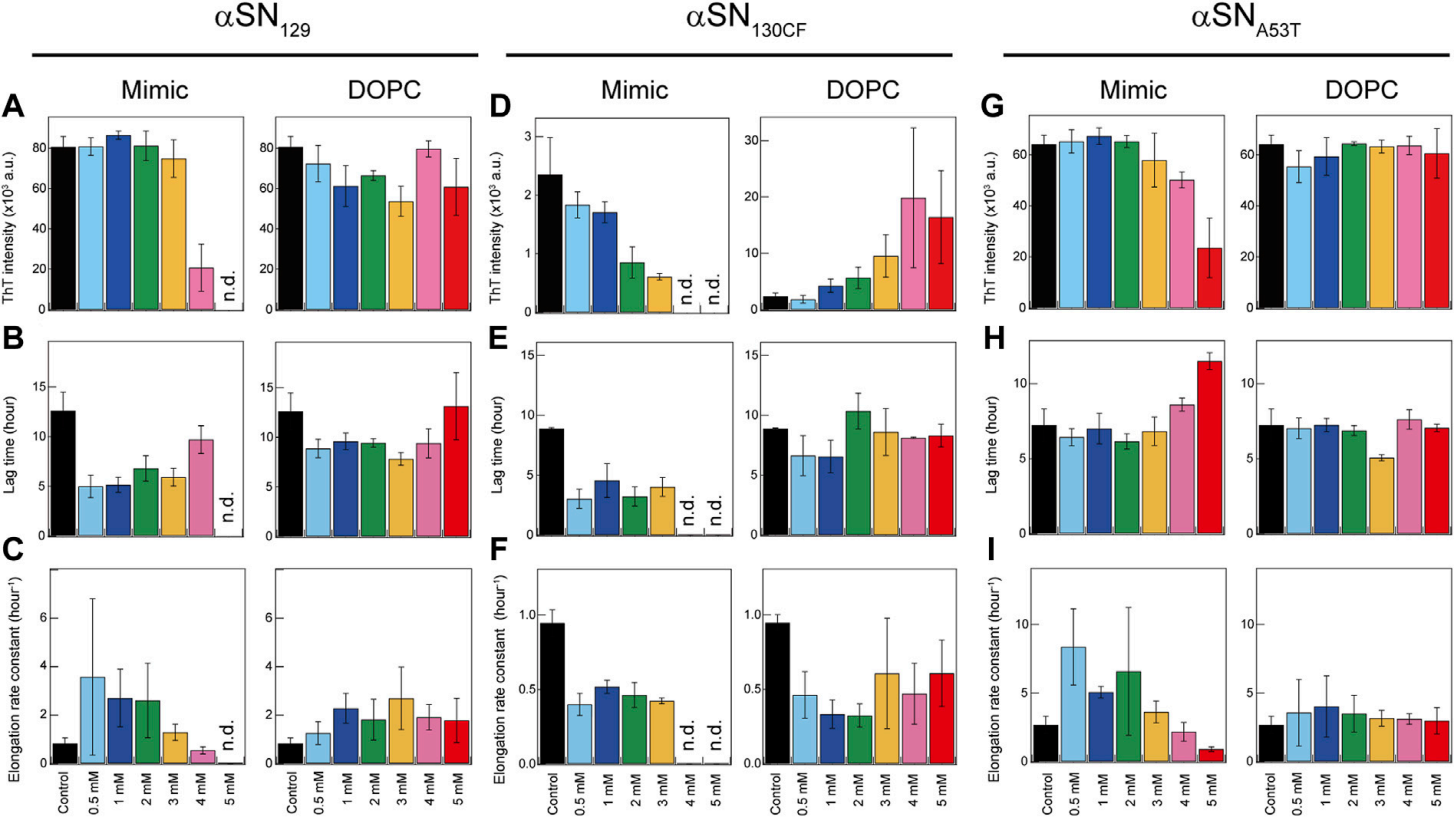
Kinetic analysis of amyloid formation of αSNs in model membranes. **(A–I)** Maximum ThT fluorescence intensities **(A**,**D**,**G)**, lag times **(B,E,H)**, and elongation rate constants **(C**,**F**,**I)** of amyloidogenesis of αSN_129_ **(A–C)**, αSN_130CF_ **(D–F)**, and αSN_A53T_ **(G–I)** in the absence and presence of Mimic (left) or DOPC membranes (right). The average values calculated from three wells in a microplate are shown with error bars reporting the standard deviation. “n.d.” denotes the concentration of lipids at which no significant increase in the ThT fluorescence intensity throughout the incubation period was observed. Various concentrations of lipids in Mimic and DOPC membranes are guided by distinct colors: black (0 mM), light blue (0.5 mM), blue (1 mM), green (2 mM), yellow (3 mM), pink (4 mM), and red (5 mM).

In the absence of the membranes, the fluorescence intensities of αSN_129_, αSN_130CF_, and αSN_A53T_ increased after a lag phase at ∼12-, ∼8-, and ∼7-h post-incubation, and reached a plateau at ∼20, ∼13, and ∼10 h after incubation, respectively (Figures 3B,F,J). These typical sigmoidal growth curves indicate nucleation-dependent amyloid formation, which was also observed for the amyloid fibrillation of αSN_WT_ in the previous result (Supplementary Figure S5) (Terakawa et al., 2018a). Moreover, the lag time reported for αSN_WT_ amyloid formation was ∼10 h, which was longer than that of αSN_A53T_ amyloid formation (Supplementary Figure S6) (Terakawa et al., 2018a). This result is consistent with those of previous reports (Conway et al., 1998; Li et al., 2001; Flagmeier et al., 2016). In addition, the post-incubation far-UV CD spectra exhibited a single negative band near 218 nm, representing *β*-sheet-rich structures of amyloid fibrils (Figures 1C,G,K). The atomic force microscopy (AFM) images revealed fibrillar aggregates of αSN_129_, αSN_130CF_, and αSN_A53T_ (Supplementary Figure S7). Notably, the maximal ThT intensity of αSN_130CF_ without lipids was markedly lower than those of αSN_129_, αSN_A53T_, and αSN_WT_ (Figures 3A,D,G; Supplementary Figure S6A), which might be explained by polymorphic amyloid formation. Indeed, secondary structure prediction showed that αSN_130CF_ amyloid fibrils formed without lipids are mostly composed of antiparallel *β*-sheets while amyloid fibrils of αSN_129_, αSN_A53T_, and αSN_WT_ contain both parallel and antiparallel *β*-sheets (Supplementary Figure S8). Collectively, the amyloid generation of all three αSN variants was verified in the absence of membranes.

The presence of Mimic membranes led to more dynamic alterations in the amyloidogenicity of αSN_129_. ThT fluorescence analysis revealed two distinct effects of Mimic membranes on fibrillation kinetics: 1) accelerated amyloid formation at lower concentrations of Mimic lipids (0.5–4 mM) with a shorter lag time and larger elongation rate constant; 2) constrained amyloid generation at higher concentrations (5 mM) with a more extended lag time and lower elongation rate constant (Figures 1B, 3A,C, left). Such results correspond to the previous finding on lipid concentration-dependent amyloidogenesis of αSN_WT_ (Terakawa et al., 2018a). As the ThT intensity might include the polymorphic aspect of amyloid fibrils hampering accurate quantification of the mass of amyloid fibrils, CD and AFM were also introduced to examine αSN_129_ amyloidogenesis. In accordance with the ThT assay results, the far-UV CD spectra at 0.5–3 mM and 4–5 mM of Mimic lipids revealed, respectively, amyloid fibrils with *β*-structures and monomers with predominant helical conformations (Figure 1C, left). The secondary structure analysis demonstrated that increases in the concentration of Mimic lipids increased and decreased the content of *α-*helix and *β*-structures, respectively. (Supplementary Figure S8). The AFM image at 5 mM of Mimic lipids further confirmed their inhibitory effects against amyloidogenesis (Figure 1D, left).

In contrast to Mimic membranes, DOPC membranes exhibited minimal effects on the amyloid fibrillation of αSN_129_. As shown in Figure 1B (right), similar nucleation-dependent sigmoidal increases in the ThT intensity were observed at all DOPC lipid concentrations (0.5–5 mM). Further kinetic analyses verified that DOPC lipids at the concentration range between 0.5 and 5 mM slightly promoted αSN_129_ amyloid formation by affecting the lag time (Figure 3B, right). Meanwhile, the elongation rate constant increased from ∼0.8 h^−1^ without lipids to ∼2 h^−1^ with 1–5 mM of DOPC lipids (Figure 3C, right). The far-UV CD spectra of αSN_129_ at all concentrations of DOPC lipids showed the formation of amyloid fibrils with *β*-sheet-rich structures after incubation (Figure 1C, right). The analysis of far-UV CD spectra confirmed the similar content of the *β*-structures at all DOPC concentrations (∼30%) (Supplementary Figure S9). It was further verified by AFM analysis at 5 mM of DOPC lipids, which exhibited clustered amyloid fibrils (Figure 1D, right). Similar minimal effects of DOPC membranes were also revealed for αSN_WT_ aggregation in the previous literature (Terakawa et al., 2018a).

Next, we investigated the effects of Mimic and DOPC membranes on αSN_130CF_ amyloid formation. The addition of 0.5–2 mM of Mimic lipids remarkably accelerated amyloid fibrillation by shortening the lag time from ∼8 to ∼4 h (Figures 1F, 3E, left). However, the elongation rate constants remain similar to that without lipids, indicating that low concentrations of Mimic lipids (0.5–2 mM) promoted αSN_130CF_ amyloidogenesis only by accelerating nucleation. Increased lipid concentrations (4–5 mM) impeded the fibrillation of αSN_130CF_, leading to no increase in the ThT intensity throughout incubation. Consistent with ThT results, far-UV CD spectra at upper range lipid concentrations showed predominant helical structures with ∼20% *α*-helix content (Figure 1G, left and Supplementary Figure S8A). Indeed, no appreciable fibrillar aggregates were detected with 5 mM of Mimic lipids (Figure 1H, left). On the other hand, the presence of DOPC membranes did not yield noticeable changes on the amyloid formation of αSN_130CF_. At all concentrations of DOPC lipids, ThT intensities increased after a lag time of ∼8–∼11 h (Figures 1F, 3E, right). Although the maximum ThT intensities at high concentrations of DOPC lipids were greater than those at low and middle concentrations of DOPC and Mimic lipids (Figure 3D, right), such discrepancy can be attributed to the polymorphic nature of amyloid fibrils which often manifest distinct structures. For example, αSN_130CF_ amyloid fibrils formed in the presence of 3 mM of DOPC lipids were mostly composed of antiparallel *β*-strands, while amyloid fibrils generated with 3 mM of Mimic lipids contained both parallel and antiparallel *β*-strands (Supplementary Figures S8,S9). Along the same lines, previous studies have reported that alterations in either lipid concentrations or liposome compositions can induce morphologically distinct amyloid fibrils (Kinoshita et al., 2017; Gaspar et al., 2021). Amyloid fibrils with different structures showed type-specific fluorescence intensity due to different binding sites of ThT on the surfaces of the amyloid fibrils (Sidhu et al., 2018). *β*-sheet-rich structures were detected at all DOPC concentrations after incubation (Figure 1G, right), with evident fibrillar aggregates formation at 5 mM of DOPC (Figure 1H, right).

Analogous to the findings for αSN_129_ and αSN_130CF_, Mimic membranes accelerated and inhibited the amyloidogenesis of αSN_A53T_ in a concentration-dependent manner. As the concentration of Mimic lipids increased from 0 to 5 mM, the elongation rate constant initially increased from ∼0.6 h^−1^ (0 mM) to ∼6 h^−1^ (0.5–2 mM), subsequently decreasing to ∼0.2 h^−1^ (5 mM) (Figures 1J, 3I, left). Interestingly, in contrast to αSN_129_ and αSN_130CF_, the lag time with 0.5–3 mM of Mimic lipids was close to that without lipids (Figure 3H, left). These results demonstrated that low concentrations of Mimic lipids (0.5–2 mM) promoted αSN_A53T_ amyloidogenesis by boosting the growth of amyloid fibrils. In addition to the decreased elongation rate constant, a significant increase in the lag time was observed at 5 mM of Mimic lipids. While some fibrillar fragments were observed at 5 mM of Mimic lipids (Figure 1L, left), the majority of αSN_A53T_ existed as helical monomers (Figures 1K, 3G, left). These results rule out the possibility that the decreased maximal ThT intensity at 5 mM of Mimic lipids resulted from the polymorphism of amyloid fibrils. On the contrary, almost no effect of DOPC membranes on the amyloid formation of αSN_A53T_ was detected. Kinetic analyses of the ThT data revealed that the lag time and elongation rate constant of αSN_A53T_ fibrillation remained steady throughout all lipid concentrations (Figures 3H,I, right). In like manner, far-UV CD spectra indicated the existence of fibrillar aggregates with *β*-sheet-rich structures at all DOPC concentrations (Figure 1K, right), which was supported by the representative AFM image in the presence of 5 mM of DOPC lipids (Figure 1L, right). The secondary structural analysis verified that the *β*-structure content of amyloid fibrils at all DOPC concentrations was ∼40% (Supplementary Figure S9D).

## Discussion

We investigated the impacts of lipid membranes on the amyloid formation of three variations of αSNs (αSN_129_, αSN_130CF_, and αSN_A53T_) with different charge states as functions of lipid component and concentration. Based on the structural, kinetic, and thermodynamic characterizations, the molecular and mechanical mechanisms of membrane-assisted acceleration and inhibition of amyloid generation were elucidated (Figures 1,3). While neutrally charged DOPC membranes showed insignificant effects on the structure and amyloidogenicity of αSNs, negatively charged Mimic membranes induced dramatic helical transitions with the dual effects of promoting and impeding amyloid aggregation depending on the membrane concentration. At low concentrations of Mimic lipids, the fibrillation of αSNs was accelerated, whereas high lipid concentrations abrogated the process. Although the dual effects of Mimic lipids were broadly applicable, low concentrations of Mimic lipids promoted the nucleation of αSN_130CF_ amyloid fibrils and elongation of αSN_A53T_ amyloid. Similar dual effects on the amyloidogenicity of αSN_WT_ were reported for other membranes with negatively charged lipids such as DOPS and DMPS (Galvagnion et al., 2015; Galvagnion et al., 2016; Jiang et al., 2018), as well as SDS (Giehm et al., 2010). Thus, these findings strongly implicate lipid concentration-dependent acceleration and inhibition of αSN amyloidogenesis under membrane-binding conditions with a net negative charge of the head groups.

**FIGURE 4:**
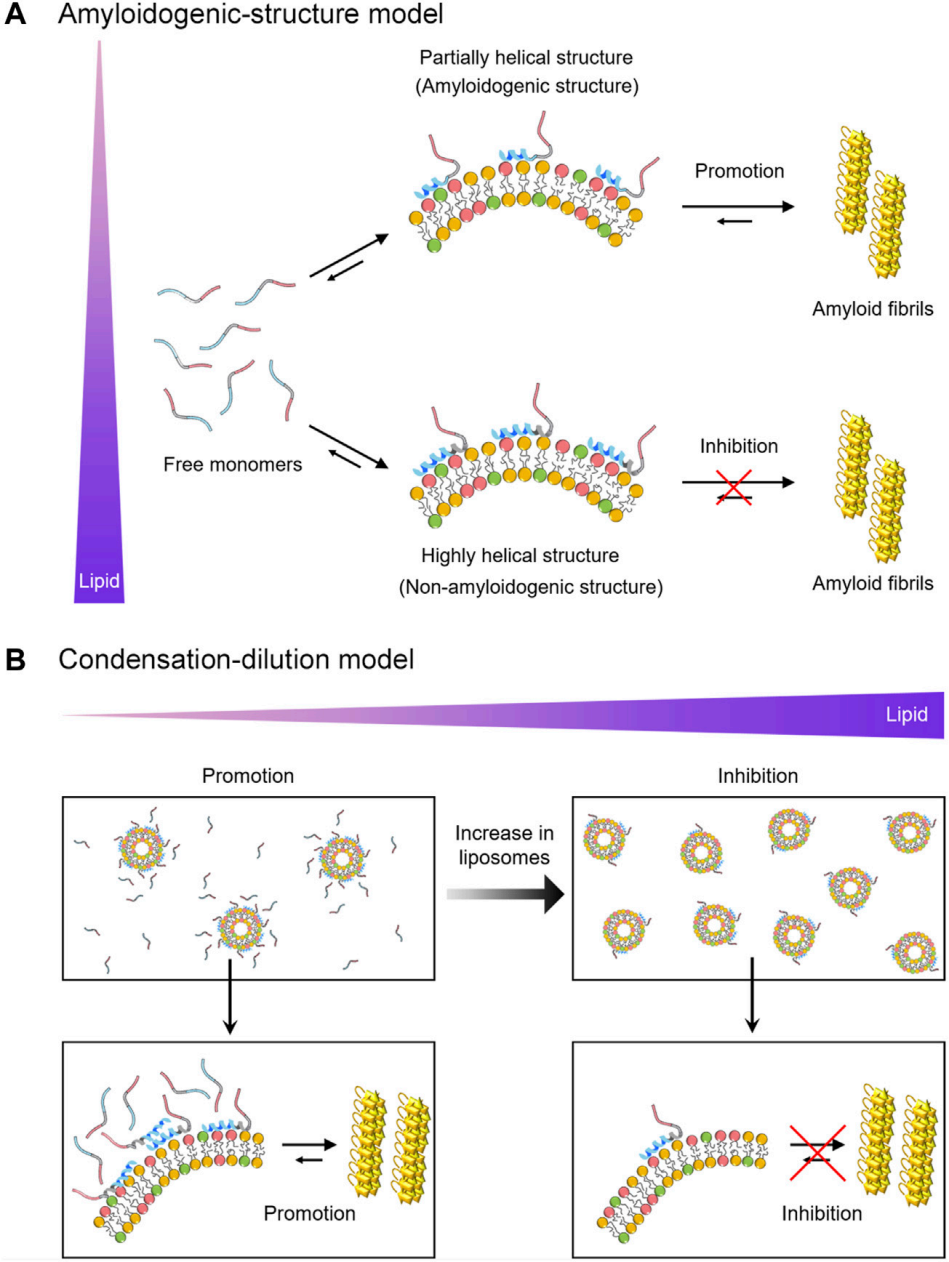
Schematic models for the dual effect of Mimic membranes on αSN amyloidogenesis. **(A,B)** Two models, the amyloidogenic structure model **(A)** and the condensation-dilution model **(B)** are schematically shown. Free monomers, partially- and highly-helical monomers in the membrane-bound forms, and amyloid fibrils are illustrated. The *N*-terminal region, the non-amyloid component region, and the *C*-terminal region of αSN are represented in blue, grey, and red, respectively. Increases in the concentration of lipids are indicated by the purple triangle.

To rationalize the dual effects of negatively charged Mimic membranes, we conceive two possible mechanisms based on the initial structure of αSN in membranes (the amyloidogenic structure) and the intermolecular affinity of αSN for membranes (condensation-dilution). The initial structure model elucidates the dual effect based on distinct structures of αSNs at varying lipid concentrations (Figure 4A). Indeed, multiple helical structures were reported for αSN on the membrane surface in previous studies (Bodner et al., 2009; Wang et al., 2010; Terakawa et al., 2018a). In line with these results, the absence of a single isodichroic point was observed in the far-UV CD spectra of three types of αSN variants at 0–5 mM of Mimic lipids (Figures 1A,E,I, left), suggesting the existence of multiple helical conformations for membrane-bound αSN. Further analyses of the CD spectra also indicated the coexistence of distinct helical structures (Supplementary Figure S3). In addition, Supplementary Figure S10 suggested that the helical content per percentage of membrane-bound αSNs showed an increasing trend with an increase in the concentration of Mimic lipids. In the absence of Mimic lipids, largely disordered αSNs slowly self-assemble into amyloid fibrils with *β*-sheet-rich structures. The addition of Mimic lipids at low concentrations triggers structural alteration from random coils to partial helical structures (Figure 4A, upper). Partial helical structures are inclined to interact with one another through helix-helix interactions, thereby facilitating nucleation for amyloidogenesis (Abedini and Raleigh, 2009; Lin et al., 2019).

Previous studies also suggested that partial helical structures are aggregation-prone and are the representative secondary structures of the key intermediates in the fibrillation pathway of αSN (Anderson et al., 2010; Ghosh et al., 2015), Aβ40 (Lin et al., 2019), hIAPP (Pannuzzo et al., 2013), and polyQ (Jayaraman et al., 2012), proposing an existence of amyloidogenic structure. Amyloidogenic structures have also been implicated in other folded proteins such as SH3 domain (Guijarro et al., 1998) and β2-microglobulin (Jahn et al., 2006). In contrast, at high concentrations of Mimic lipids, αSNs adopt prominent helical structures with an exceptionally low aggregation propensity (Figure 4A, lower). These highly helical non-amyloidogenic structures were analogously observed in Aβ40, Aβ42, and αSNs at high concentrations of alcohols (e.g., 40% TFE and 50% HFIP) (Crescenzi et al., 2002; Anderson et al., 2010; Lin et al., 2019). Accordingly, αSNs in bulk aqueous solution would take time to form a nucleus with an amyloidogenic structure in a conformational ensemble. It should be noted that a possible binding model such as the insertion of αSNs into lipid bilayers is excluded from Figure 4 for simplification.

Condensation-dilution model explains the mechanism of the dual effect on the basis of the thermodynamic binding affinity (Figure 4B). At low lipid concentrations, αSN binds multiply with Mimic membranes, which leads to increased local concentrations of αSN (Figure 4B, left). Thus, concentrated αSN will be sufficient to facilitate nucleation for amyloid fibrillation. The growth process can be expedited by elongation with the addition of neighboring monomers around fibril seeds. Similar surface-induced enhancement of the local protein concentration to boost amyloid fibrillation was also observed for Aβ and β2-microglobulin (Linse et al., 2007; Cabaleiro-Lago et al., 2010). In addition, although the quantity of free αSN in bulk solution was also important for amyloidogenesis in membrane environments, the increases in the local concentration of αSNs to accelerate amyloid formation at low lipid concentrations overwhelms the decreased concentration of free αSN slowing down amyloid generation, and, thus, leads to the acceleration of amyloid fibrillation. However, at high lipid concentrations, αSNs will be spread across discrete liposomes and their membranes, leading to diluted local concentrations of αSN. As a result, the amount of αSNs in each liposome and bulk water decrease significantly. This, in turn, interferes with efficient nucleation and elongation, causing the prevention of amyloid formation (Figure 4B, right). Along the same lines, our results demonstrated that significant inhibitory effects of Mimic lipids were observed at the concentrations where ∼80% of αSNs were bound to Mimic lipids (Supplementary Figure S11). ∼20% of free αSNs in bulk solution were not sufficient for efficient nucleation and elongation. When the lag time of αSNs amyloid fibrillation was plotted as a function of the population of membrane-bound αSNs, a V-shaped dependence was observed with a minimum at approximately 40% (Supplementary Figure S12A). These results suggested that the shortest lag time for amyloidogenesis of all types of αSN was achieved when approximately 40% monomers were bound to membranes of Mimic lipids. On the other hand, no clear correlation was observed for the elongation rate constant. A moderate negative correlation between the elongation rate constant and the population of membrane-bound αSNs (*R* = −0.67 and *p* = 0.02) was observed in higher populations of membrane-bound αSNs; however, no correlation (*R* = 0.15 and *p* = 0.64) was detected in lower populations of membrane-bound αSNs. This result suggested that the dual effect is prominent for the lag time in relation to nucleation, and, the elongation rate constant might be variable depending on the type of αSN (Supplementary Figure S12B). In addition, the condensation-dilution model also illustrates the aggregation of Aβ at the various concentration of cationic polystyrene nanoparticles (Cabaleiro-Lago et al., 2010).

Biological membranes have shown their capability to modulate folding, aggregation, and the function of αSN (O'Leary and Lee, 2019). Binding affinity of αSN for membranes is influenced not only by the properties of lipid bilayers such as the net charge and curvature (Middleton and Rhoades, 2010), but also by mutations and post-translational modifications including phosphorylation (Kuwahara et al., 2012) and N-terminal acetylation (Runfola et al., 2020). In the current study, we revealed that the binding affinity of αSNs to Mimic membranes decreased in the order αSN_130CF_, αSN_129_, αSN_WT_, and αSN_A53T_. This indicates that the removal of negatively charged residues between positions 130 and 140 increases the membrane binding affinity, whereas repulsive electrostatic interactions between negatively charged C-terminal domain of αSN and Mimic membranes decrease the intermolecular affinity. Considering that the large energy gain for αSN upon membrane binding is derived from electrostatic interactions between the positively charged NTR of αSN and negatively charged membranes, electrostatic forces are fundamental for αSN-membrane interactions. Increased affinity for membranes with an additional positive charge in the NTR of E46K further supports the importance of electrostatic contributions (Stockl et al., 2008). In addition, we speculate that a point mutation in the NTR like αSN_A53T_ might impair favorable electrostatic interactions with membranes, which attenuates the overall affinity. Furthermore, Mimic lipids with a strong binding affinity (*K*_d_ = ∼200 nM) exert dual effects on amyloid formation of αSN_WT_. In contrast, DOPC lipids with a weak binding affinity (*K*_d_ = n.d.) showed a minimal effect on amyloidogenesis. Thus, we consider that the binding affinity between αSN and membranes plays a key role in modulating the amyloidogenicity and amyloidogenesis of αSN.

In line with present results, a recent study reported that the addition of calcium ions significantly increases αSN’s propensity to interact with negatively charged membranes by reducing repulsive electrostatic interactions of negatively charged C-terminal regions with membranes (Lautenschlager et al., 2018). Along the same lines, the higher affinity of αSN_130CF_ can be attributed to possible contacts of neutralized 10 residues with membranes via non-polar interactions. It should be also noted that the minimal concentration of Mimic lipids for blocking fibrillation (αSN_130CF_: 4 mM; αSN_129_ = αSN_WT_: 5 mM; αSN_A53T_: > 5 mM) mostly followed the reverse order of the binding affinity, which further supports the condensation-dilution model. These data also imply that the C-terminal region might induce alterations in membrane-induced αSN amyloidogenesis by adjusting the binding affinity. Overall, the relative molar ratio of αSN to the lipid concentration is a decisive parameter of amyloid generation in presynaptic vesicles.

Phase diagrams are highly valuable for a comprehensive understanding biological and pathogenic phase transitions including protein aggregation (Lin et al., 2014; Lin et al., 2016; Terakawa et al., 2018a; Terakawa et al., 2018b; Lin et al., 2019; Gee et al., 2020; Ivanova et al., 2021). To illustrate membrane-induced amyloidogenesis of αSNs, we constructed conceptual phase diagrams of αSN_130CF_, αSN_129_, and αSN_A53T_ depending on the concentrations of αSN and Mimic lipids (Supplementary Figure S13). Each αSN displays soluble-to-insoluble phase transition following thermodynamic equilibration. Displaying amyloid-forming regions of αSN_130CF_, αSN_129_, and αSN_A53T_ at 50 μM respectively at 0–4, 0–3, and 0–5 mM of Mimic lipids demonstrate the minimal concentration of Mimic lipids required to impede the fibrillation process. Further elevations in the lipid concentration beyond the amyloid-forming region may increase the solubility of αSNs, thereby preventing their aggregation.

Understanding of context-dependent kinetics and amyloidogenicity of αSN is essential for overcoming synucleinopathies with cytotoxic aggregation in cells. Depending on its cellular localization and neighboring components, the amyloid fibrillation of αSN will be both faster and slower in bulk solution than in biological membranes, including presynaptic vesicles, due to the dual effect. As the dual effect based on two possible models suggests, αSN amyloid fibrillation is subjected to acceleration or inhibition depending on the structural state of αSN and its relative affinity for membranes. In summation, the modulation of amyloidogenesis is governed by various conditions that regulate electrostatic interactions between αSN and membranes through a favorable enthalpic contribution. The combination of our results and previous data implies that biphasic modulation of the amyloidogenesis of αSN is a generic feature of negatively charged membranes. Both “amyloidogenic structure” and “condensation-dilution” models are necessary to explain the dual effect of negatively charged lipid membranes on αSN amyloidogenesis. Further studies at lipid concentrations higher than 5 mM will advance understanding of the dual effect of membranes on αSNs amyloid fibrillation. Moreover, varying αSN concentrations with a fixed concentration of lipids will provide an alternative opportunity to modulate the lipid/protein ratio. Thus, a series of further experiments at various concentrations of αSNs will make the concept of the dual effect more solid.

αSN_WT_ and its aggregated states have been reported to induce membrane disruption (van Rooijen et al., 2009; Reynolds et al., 2011; Fusco et al., 2017; Surguchov et al., 2017; Iyer and Claessens, 2019), leading to the increased influx of Ca^2+^ into cells. As mitochondria are highly susceptible to the abnormal ionic strength, Ca^2+^ dysregulation can induce an apoptotic cascade, and, subsequently cell dysfunction and death (Duchen, 2000; Angelova et al., 2016). Pore formation has been considered to be a major mechanism responsible for αSN-induced membrane disruption (Surguchov et al., 2017). Lansbury and coworkers proposed that annular protofibrils of αSN might incorporate into membranes for the pore formation (Lashuel et al., 2002). Highly helical αSN monomers have also been observed to form ion channel-like pores in membranes (Zakharov et al., 2007). As demonstrated in the current study, the mutations of αSN alter membrane binding properties and aggregation behaviors on the membrane surface. Understanding of how αSN_WT_ and its variant distinctively influence membrane integrity depending on the mutation of αSN and type of lipids will be an interesting topic for a future study.

## Data Availability

The raw data supporting the conclusion of this article will be made available by the authors, without undue reservation.
