# Supplementary material for: Dual Effects of Presynaptic Membrane Mimetics on α-Synuclein Amyloid Aggregation
Source: Front Cell Dev Biol. 2022 Jun 7;10:707417. doi: 10.3389/fcell.2022.707417 (PMC9209734; doi:10.3389/fcell.2022.707417)
Supplement: Supplementary file 1 [file DataSheet1.pdf]

Supplementary Information
for
Dual effects of presynaptic membrane mimetics on α-synuclein amyloid aggregation

Yuxi Lin[1,2*], Dai Ito[3], Je Min Yoo[4], Mi Hee Lim[5], Wookyung Yu[3,6], Yasushi Kawata[7], Young-Ho Lee[1,2,8,9,10*]

[1]Research Center for Bioconvergence Analysis, Korea Basic Science Institute (KBSI), Ochang, Chungbuk 28119, Republic of Korea

[2]Institute for Protein Research, Osaka University, Yamadaoka 3-2, Suita, Osaka 565-0871, Japan

[3]Department of Brain and Cognitive Science, Daegu Gyeongbuk Institute of Science and Technology (DGIST), Daegu, 42988, Republic of Korea

[4]BIOGRAPHENE, 555 W. 5th St., Los Angeles, California 90013, United States.

[5]Department of Chemistry, Korea Advanced Institute of Science and Technology (KAIST), Daejeon 34141, Republic of Korea

[6]Core Protein Resources Center, Daegu Gyeongbuk Institute of Science and Technology (DGIST), Daegu, 42988, Republic of Korea

[7]Department of Chemistry and Biotechnology, Graduate School of Engineering, Tottori University, Tottori 680-8550, Japan

[8]Bio-Analytical Science, University of Science and Technology (UST), Daejeon 34113, Republic of Korea

[9]Graduate School of Analytical Science and Technology (GRAST), Chungnam National University (CNU), Daejeon 34134, Republic of Korea

[10]Research Headquarters, Korea Brain Research Institute (KBRI), Daegu 41068, Republic of Korea

*To whom correspondence should be addressed: mr0505@kbsi.re.kr (Y.-H.L.) and linyuxi@kbsi.re.kr (Y.L.)

1. Supplementary Materials and Methods

Dynamic light scattering

Dynamic light scattering (DLS) measurements of Mimic and DOPC small unilamellar vesicles (SUVs) were performed on a Zetasizer NanoZS (Malvern Panalytical, UK) at 25 ^{o}C. Sample solutions (100 µL) containing 1 mM lipids, 20 mM sodium phosphate buffer (pH 7.4), and 100 mM NaCl were loaded into a disposable micro cuvette (Model ZEN0040). The hydrodynamic radius (R_H) was calculated using the Zetasizer software (Malvern Panalytical, ver. 7.12).

Atomic force microscopy

After incubating αSN monomers with Mimic and DOPC membranes at 5 mM lipids, sample drops of 50 µM αSNs were deposited on freshly cleaved mica plates. Following 1 min, the remaining solution was blown off with compressed air and further air-dry. Atomic force microscopy images were acquired using a Digital Instruments Nanoscope IIIa scanning microscope (Veeco, Santa. Barbara, CA) with a Si microcantilever.

Circular dichroism spectroscopy

Circular dichroism (CD) experiments were carried out on a JASCO J820 spectrophotometer (Tokyo, Japan) at 37 ^{o}C. The far-UV CD spectra of αSNs in 20 mM sodium phosphate buffer (pH 7.4) containing 100 mM NaCl and various concentrations of lipids of DOPC and Mimic membranes were recorded using a quartz cuvette with a 0.1-mm path length. After subtracting the solvent background, CD signals were presented as the mean residue ellipticity (deg·cm^{2}· dmol^{-1}). The content of secondary structures was

predicted using the BeStSel algorithm (Micsonai et al., 2015).

Determination of the population of membrane-bound αSNs.

To calculate the population of membrane-bound αSNs, free αSN (FαSN) is assumed to bind to multiple lipid molecules ($\mathrm{Lipid_N}$) with a K_d based on the following scheme (Galvagnion et al., 2015;Terakawa, Lee, and Kinoshita et al., 2018):

$$\mathrm{F\alpha SN} \ + \ \mathrm{Lipid_N} \ \leftrightarrow \ \mathrm{B\alpha SN(Lipid)_N}$$

where N represents the number of lipids interacting with one αSN.

Thus, K_d is defined by the following equation:

$$K_d = \frac{[\mathrm{F\alpha SN}][\mathrm{Lipid_N}]}{[\mathrm{B\alpha SN(Lipid)_N}]}$$

Equation S1

where [FαSN], [$\mathrm{Lipid_N}$], and [$\mathrm{B\alpha SN(Lipid)_N}$] represent the concentration of free αSN, lipid molecules responsible for binding to one αSN, and αSN:lipid complex, respectively.

The total concentration of αSN ($[\alpha SN]_t$) and lipid ($[Lipid]_t$) are described as follows:

$$[\alpha\mathrm{SN}]_\mathrm{t} = [\mathrm{F}\alpha\mathrm{SN}] \ + \ [\mathrm{B}\alpha\mathrm{SN}(\mathrm{Lipid})_\mathrm{N}]$$

Equation S2

$$[\mathrm{Lipid}]_\mathrm{t} = \mathrm{N}([\mathrm{Lipid}_\mathrm{N}] \ + \ [\mathrm{B}\alpha\mathrm{SN}(\mathrm{Lipid})_\mathrm{N}])$$

Equation S3

Equation S1 was substituted with equations S2 and S3 to yield the following equation:

$$[\mathrm{B}\alpha\mathrm{SN}(\mathrm{Lipid})_\mathrm{N}] = \frac{\left([\alpha\mathrm{SN}]_\mathrm{t} + \frac{[\mathrm{Lipid}]_\mathrm{t}}{\mathrm{N}} + K_d\right) - \sqrt{\left([\alpha\mathrm{SN}]_\mathrm{t} + \frac{[\mathrm{Lipid}]_\mathrm{t}}{\mathrm{N}} + K_d\right)^2 - \frac{4[\alpha\mathrm{SN}]_\mathrm{t}[\mathrm{Lipid}]_\mathrm{t}}{\mathrm{N}}}}{2}$$

Equation S4

The population of membrane-bound αSNs (P_b) is defined as follows:

$$\mathrm{P_b} = \frac{[\mathrm{B}\alpha\mathrm{SN}(\mathrm{Lipid})_\mathrm{N}]}{[\alpha\mathrm{SN}]_\mathrm{t}}$$

Equation S5

Equation S4 was substituted into S5 to give the following equation:

$$P_b = \frac{\left([\alpha SN]_t + \frac{[Lipid]_t}{N} + K_d\right) - \sqrt{\left([\alpha SN]_t + \frac{[Lipid]_t}{N} + K_d\right)^2 - \frac{4[\alpha SN]_t[Lipid]_t}{N}}}{2[\alpha SN]_t}$$

Equation S6

where $[\alpha SN]_t$ was 50 μM. The values of K_d and N were obtained from the ITC measurements. The N values of αSN_{WT}, αSN_{129}, αSN_{130CF}, and αSN_{A53T} were 47.6, 50, 55.6, and 52.6, respectively.

Determination of the helix content per percentage of bound αSN

The helix content per percentage of bound αSN (H_b) was calculated using the following equation:

$$H_b = \frac{H_t - H_i * (1 - P_b)}{P_b}$$

Equation S7

where H_i is the helix content of αSN in the absence of Mimic lipids. H_t and P_b are the total helix content and the population of bound αSN at the desired concentration of Mimic lipids, respectively. H_t and H_i were obtained by analyzing the CD spectra using the BeStSel algorithm (Micsonai et al., 2015).

2. Supplementary Figures

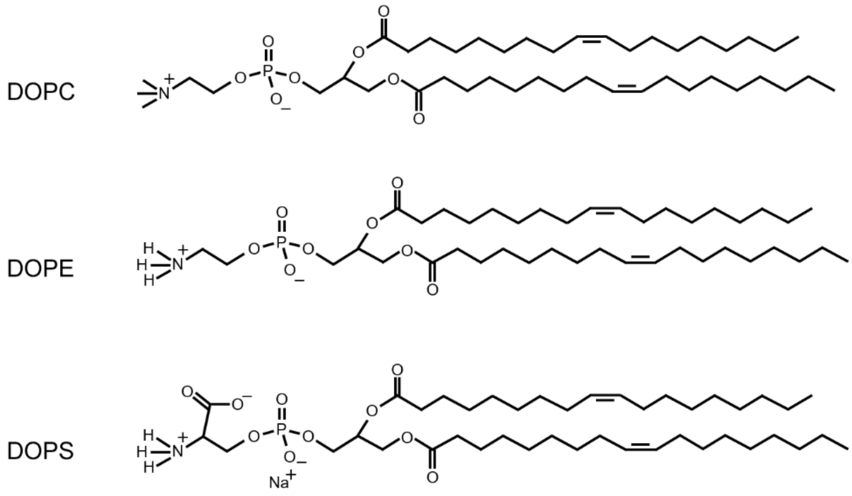

Figure S1. Chemical structures of phospholipids. Three types of phospholipids are shown: 1,2-dioleoyl-sn-glycero-3-phosphocholine (DOPC), 1,2-dioleoyl-*sn*-glycero-3-phosphoethanolamine (DOPE), and 1,2-dioleoyl-*sn*-glycero-3-phospho-l-serine (DOPS).

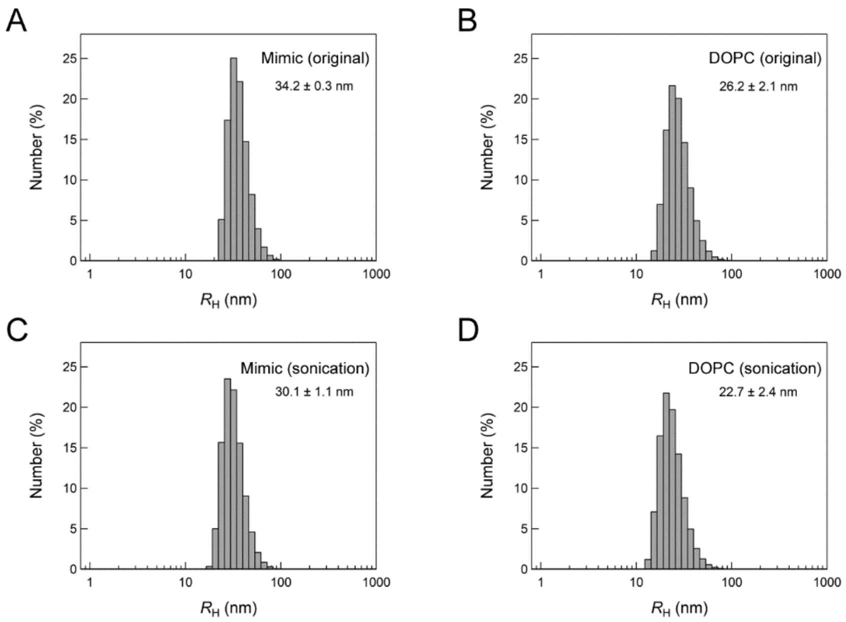

Figure S2. Hydrodynamic radii of the two types of liposomes. (**A - D**) R_H distribution of Mimic (**A** and **C**) and DOPC (**B** and **D**) liposomes before (**A** and **B**) and after (**C** and **D**) incubation with ultrasonication. The average and error values obtained from triplicate measurements of the same sample are displayed.

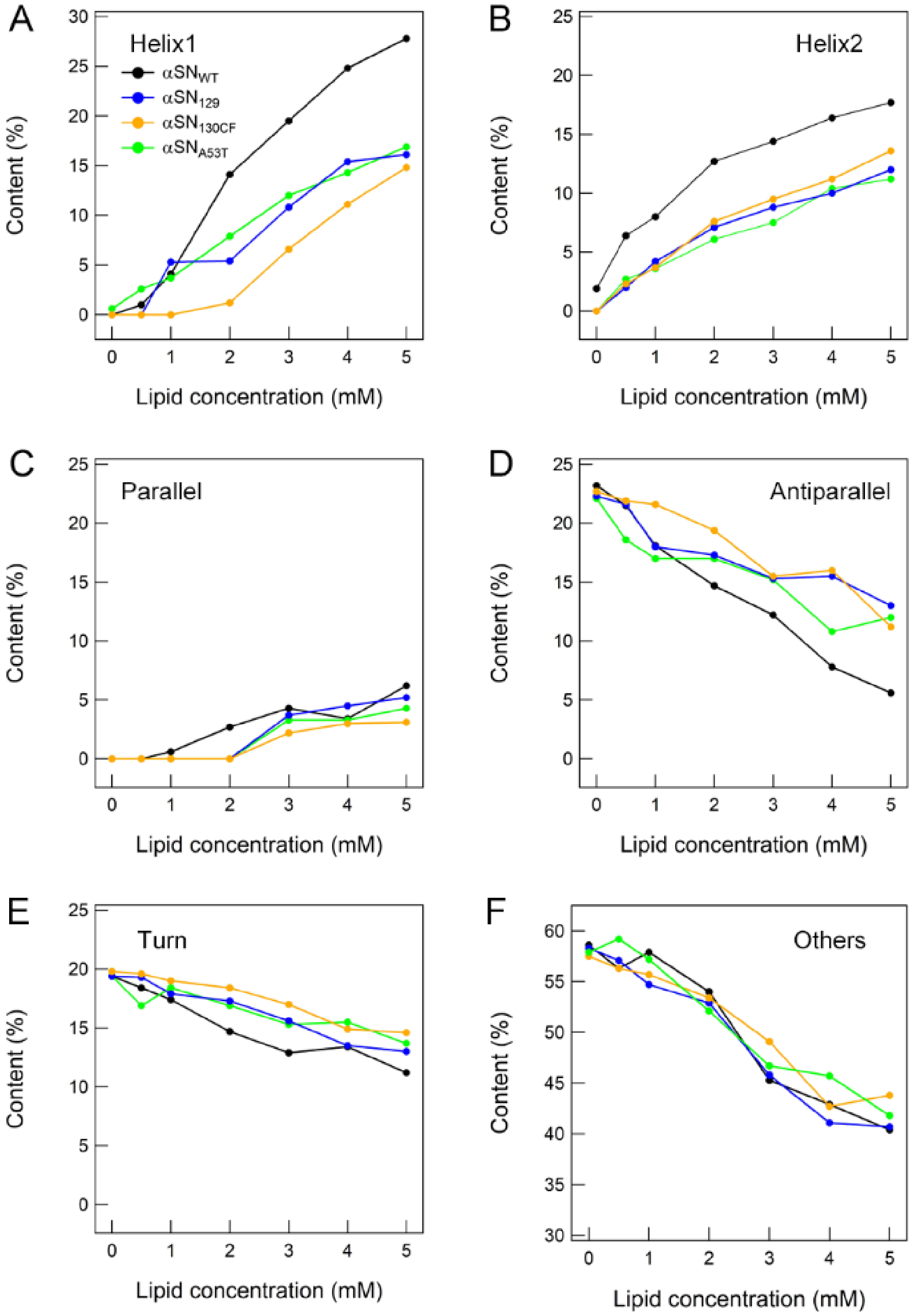
A
Helix1
αSN_WT
αSN_129
αSN_130CF
αSN_A53T
Content (%)
Lipid concentration (mM)
B
Helix2
C
Parallel
D
Antiparallel
E
Turn
F
Others

Figure S3. Contents of the secondary structure of αSNs at the various concentrations of lipids of Mimic membranes. (**A**-**F**) Contents of helix1 (**A**), helix2 (**B**), parallel β-sheet (**C**), antiparallel β-sheet (**D**), turn (**E**), and others (**F**) are plotted as a function of the concentration of Mimic lipids. Calculated contents of αSNs are displayed in distinct colors: αSN_{WT} (black), αSN_{129} (blue), αSN_{130CF} (yellow), and αSN_{A53T} (green). Results of αSN_{WT} were reproduced with modifications from our previous study (Terakawa, Lee, and Kinoshita et al., 2018).

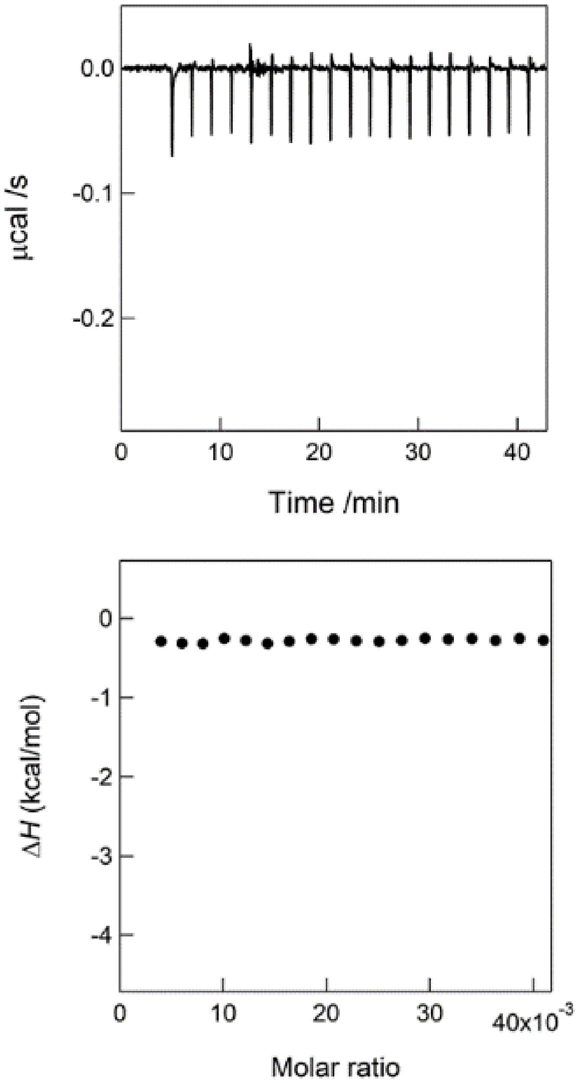

Figure S4. Characterization of the interactions between αSN$_{WT}$ and DOPC membranes. ITC thermogram (upper) and binding isotherm (lower) obtained by titrating αSN$_{WT}$ to DOPC membranes are shown.

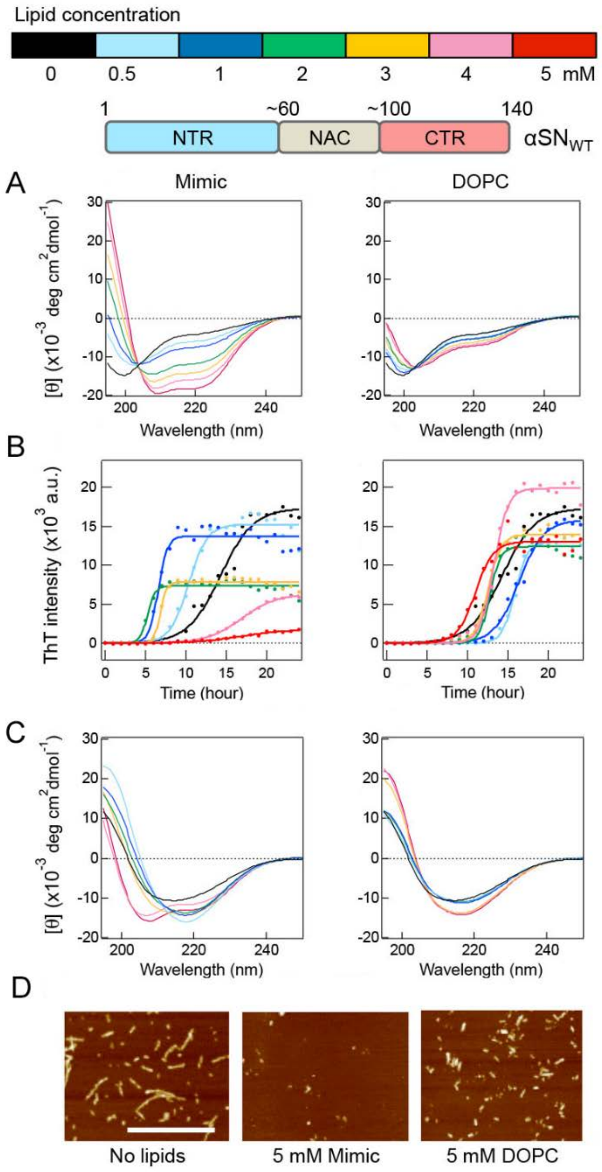

Figure S5. Effects of model membranes on the structure and amyloid formation of αSN$_{WT}$. (**A-D**) Conformational transitions and fibrillation kinetics of αSN$_{WT}$ in the absence and presence of Mimic (left) and DOPC membranes (right). Far-UV CD spectra of αSN$_{WT}$ before (**A**) and after (**C**) incubation were acquired. (**B**) Fibrillation kinetics of αSN$_{WT}$ were monitored using the ThT fluorescence assay. Solid lines represent the fit curves. Schematic representations of αSN$_{WT}$ are shown above the corresponding data.

The *N*-terminal region (NTR), the non-amyloid β component (NAC) region, and the *C*-terminal region (CTR) are colored in blue, grey, and red, respectively. Various concentrations of lipids in Mimic and DOPC membranes are denoted with distinct colors: black (0 mM), light blue (0.5 mM), blue (1 mM), green (2 mM), yellow (3 mM), pink (4 mM), and red (5 mM). (**D**) AFM images were taken for the samples of αSN_{WT} incubated in the absence (left) and presence of 5 mM Mimic (left) or DOPC (right) lipids. The white scale bars indicate 500 nm. Results were reproduced with modifications from our previous study (Terakawa, Lee, and Kinoshita et al., 2018).

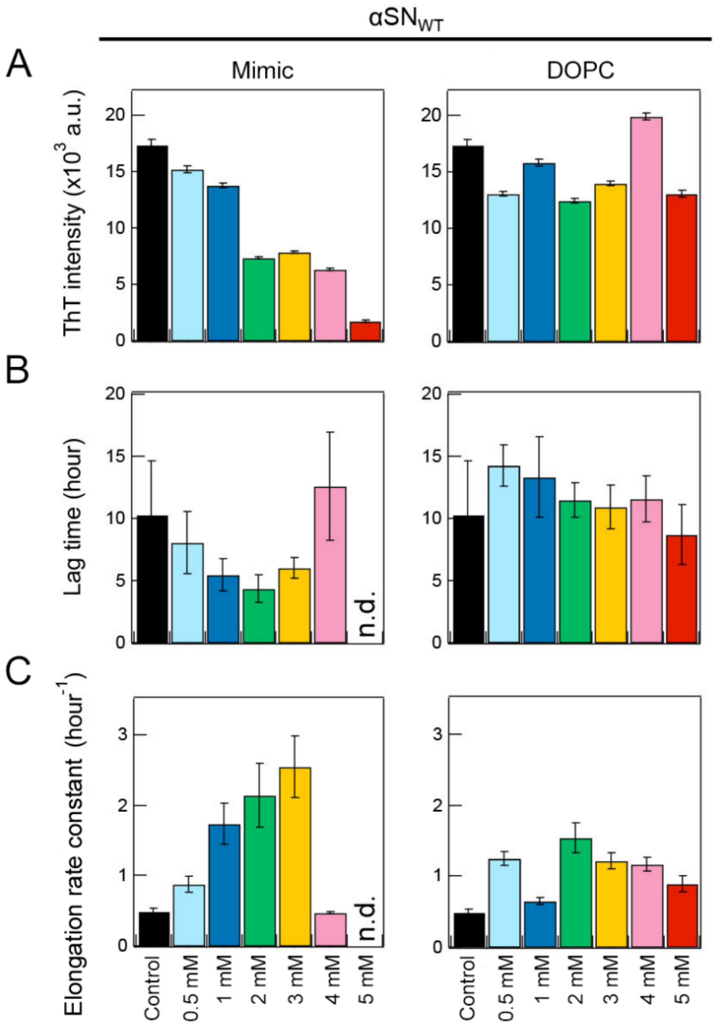

Figure S6. Kinetic analysis of amyloid formation of αSN$_{WT}$ in model membranes. (**A-I**) Maximum ThT fluorescence intensities (**A**), lag times (**B**), and elongation rate constants (**C**) of amyloidogenesis of αSN$_{WT}$ in the absence and presence of Mimic (left) or DOPC membranes (right). "n.d." denotes the concentration of lipids at which no significant increase in the ThT fluorescence intensity throughout the incubation period was observed. Various concentrations of lipids in Mimic and DOPC membranes are guided by distinct colors: black (0 mM), light blue (0.5 mM), blue (1 mM), green (2 mM),

yellow (3 mM), pink (4 mM), and red (5 mM). Results were reproduced with modifications from our previous study (Terakawa, Lee, and Kinoshita et al., 2018).

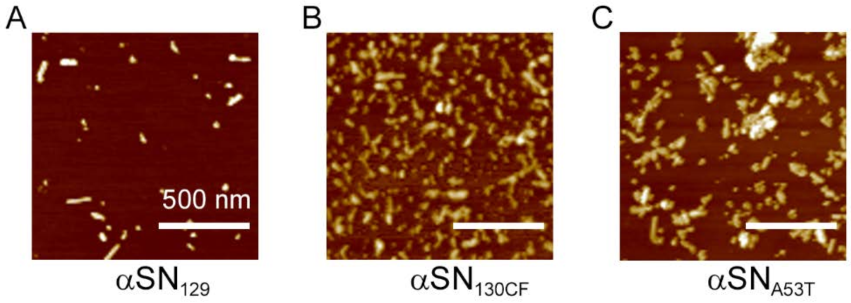

Figure S7. Morphological characterization of αSNs amyloid fibrils. (**A**-**C**) AFM micrographs of amyloid fibrils of αSN_{129} (**A**), αSN_{130CF} (**B**), and αSN_{A53T} (**C**) formed after incubation without lipid membranes. The white scale bars indicate 500 nm.

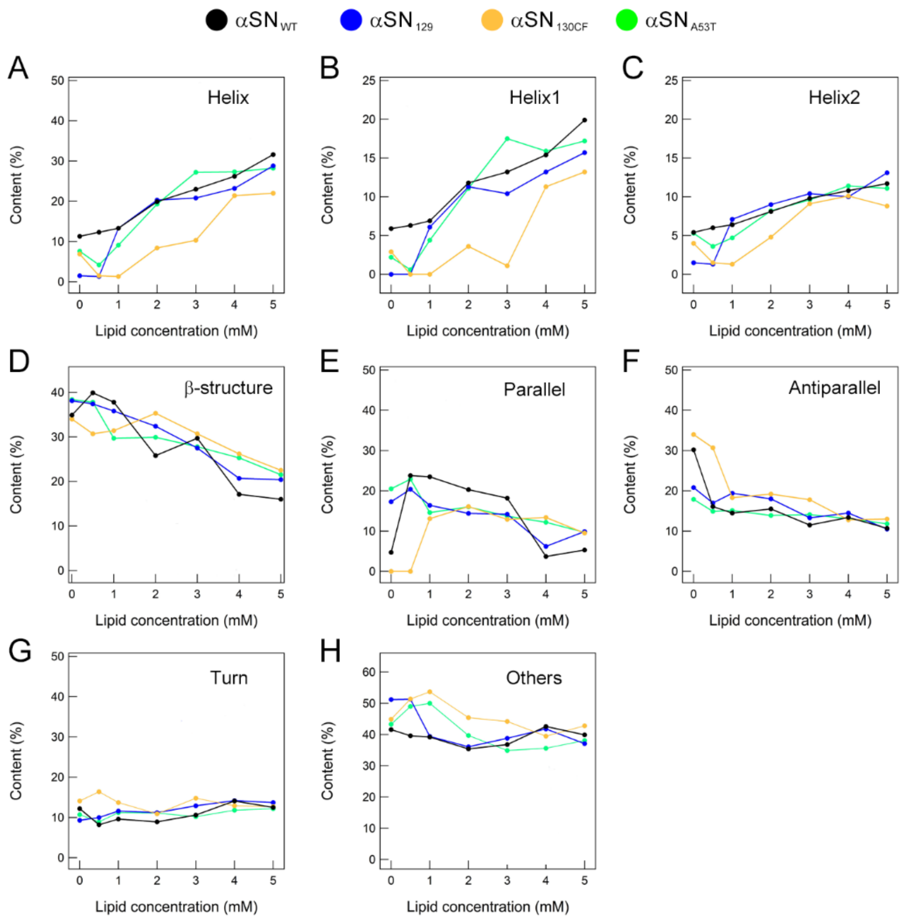

Figure S8. Contents of the secondary structures of αSNs after incubation with various concentrations of lipids of Mimic membranes. (**A-H**) Contents of helix (**A**), helix1 (**B**), helix2 (**C**), β-structure (**D**), parallel β-strand (**E**), antiparallel β-strand (**F**), turn (**G**), and others (**H**) are plotted as a function of the concentration of Mimic lipids. The helix content is the sum of the helix1 and helix2 contents. The β-structure content represents the sum of the contents of parallel and antiparallel β-strands. The calculated contents of αSNs are displayed in distinct colors: αSN$_{WT}$ (black), αSN$_{129}$ (blue), αSN$_{130CF}$ (yellow), and αSN$_{A53T}$ (green).

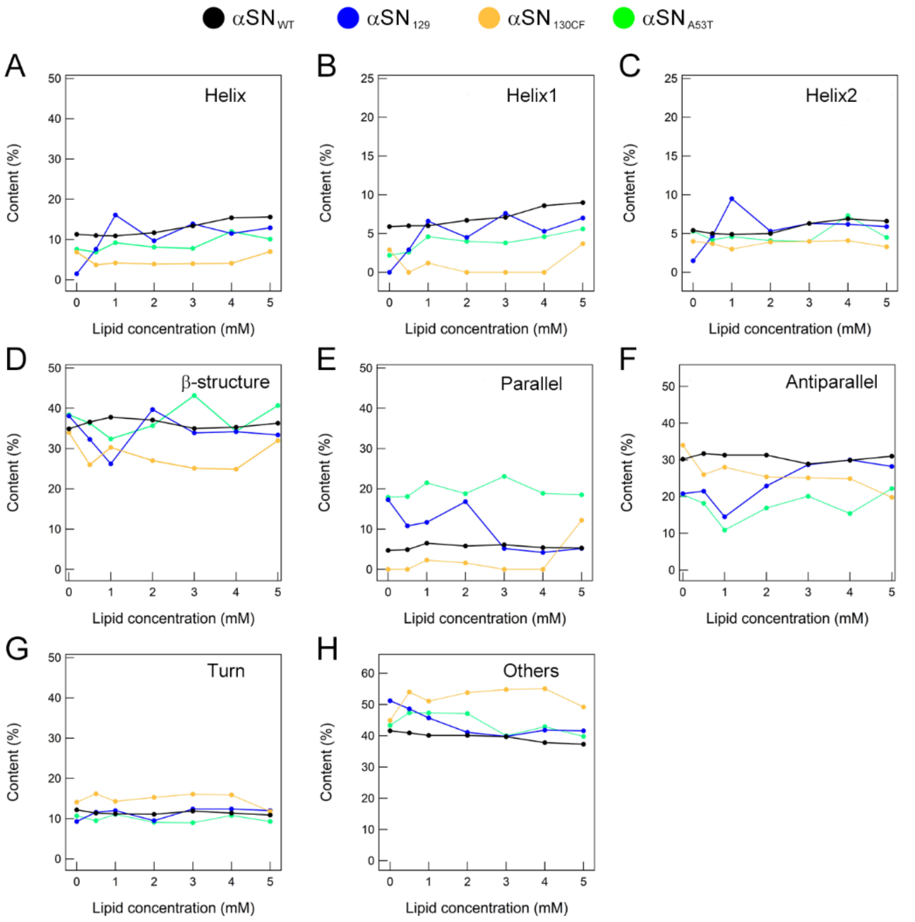

Figure S9. Contents of the secondary structure of αSNs after incubation with various concentrations of lipids of DOPC membranes. (**A**-**H**) Contents of helix (**A**), helix1 (**B**), helix2 (**C**), β-structure (**D**), parallel β-strand (**E**), antiparallel β-strand (**F**), turn (**G**), and others (**H**) are plotted as a function of the concentration of DOPC lipids. The helix content is the sum of the helix1 and helix2 contents. The β-structure content represents the sum of the contents of parallel and antiparallel β-strands. The calculated contents of αSNs are displayed in distinct colors: αSN_{WT} (black), αSN_{129} (blue), αSN_{130CF} (yellow), and αSN_{A53T} (green).

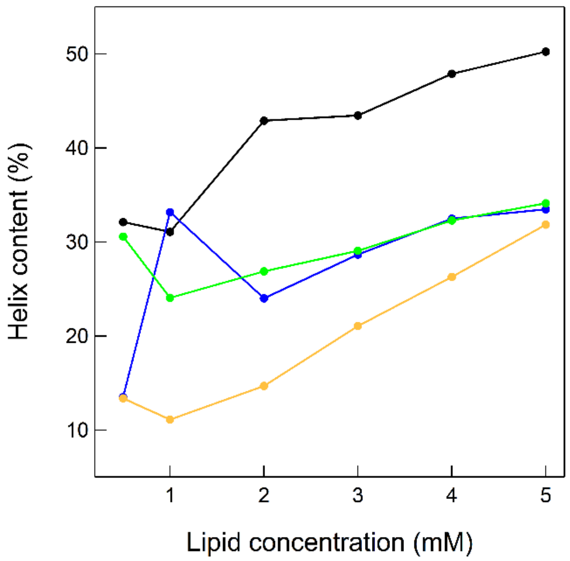

Figure S10. Helix content per percentage of bound αSNs at various concentrations of lipids of Mimic membranes. Helix content per percentage of bound αSN_{WT} (black), αSN_{129} (blue), αSN_{130CF} (orange), and αSN_{A53T} (green) are plotted as a function of the concentration of Mimic lipids.

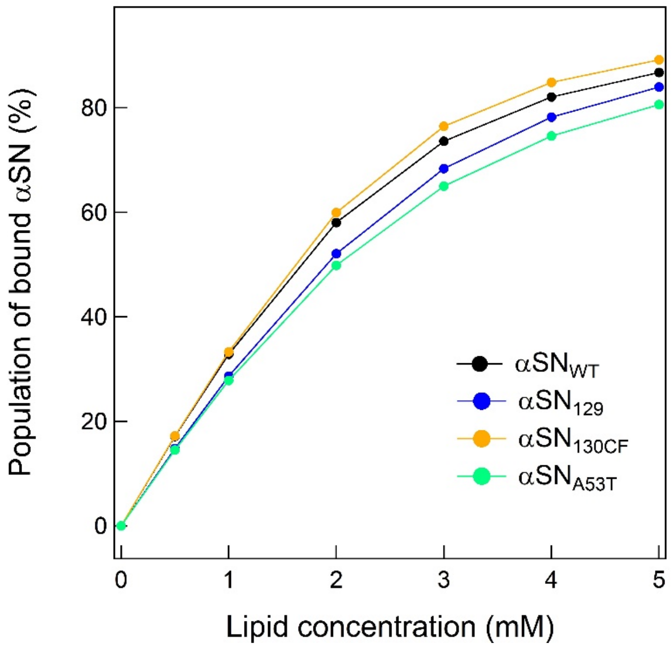

Figure S11. Population of bound αSNs at various concentrations of lipids of Mimic membranes. The population of bound αSN_{WT} (black), αSN_{129} (blue), αSN_{130CF} (orange), and αSN_{A53T} (green) are plotted as a function of the concentration of Mimic lipids.

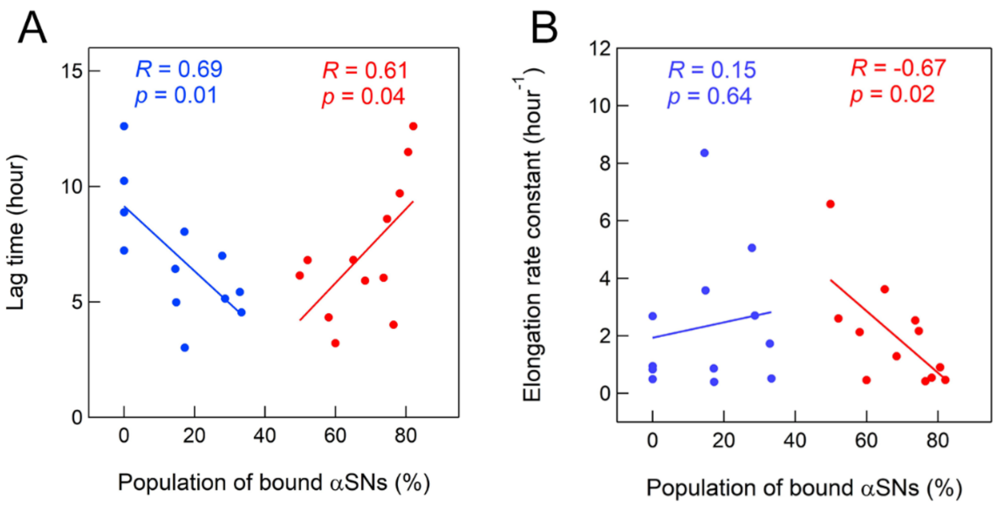

Figure S12. Correlation between the kinetic parameters of αSNs amyloidogenesis and the membrane-bound population of αSN monomers. (**A** and **B**) Linear regression between either the lag time (**A**) or elongation rate constant (**B**) of αSNs amyloid fibrillation and the population of membrane-bound αSNs (*i.e.*, αSN_{WT} and its three variants). The data corresponding to the acceleration (blue) and inhibition (red) of amyloid fibrillation are shown with different colors. All data of αSN_{WT}, αSN_{129}, αSN_{130CF}, and αSN_{A53T} were used for analyses. The population of membrane-bound αSNs is indicated as the population of bound αSNs in the X-axis. Pearson's correlation coefficient (R) and p-value are indicated in **A** and **B**. Statistical significance was set at $p < 0.05$.

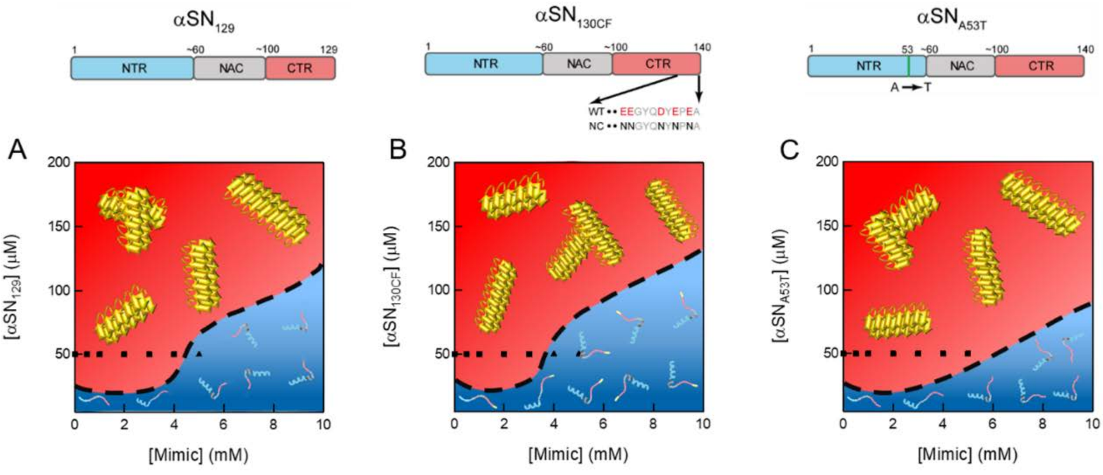

Figure S13. Macroscopic phase diagrams of αSNs aggregation in Mimic membranes. (**A**−**C**) Schematic representation for phase diagrams of amyloid formation of αSN_{129} (**A**), αSN_{130CF} (**B**), and αSN_{A53T} (**C**) in the presence of Mimic membranes. Colors and symbols represent the molecular species and colloidal states: soluble monomers (blue region and ▲) and mature amyloid fibrils (red region and ■). Symbols (■ and ▲) were plotted against the lipid concentration that was used for experiments. Cartoons for individual molecular species (largely-disordered monomer (bulk solution), highly-helical monomer (membrane-bound form), and amyloid fibril) are illustrated. Three distinct regions of αSN monomers are displayed in different colors: the N-terminal region (blue), the non-amyloid component region (grey), and the C-terminal region (red). Yellow color of αSN_{130CF} in the phase diagram indicates the region from E130 to A140 where negatively-charged residues are neutralized. Broken black lines at each phase diagram indicate conceptual solubility curves. The region at which concentrations of Mimic lipids are larger than 5 mM is conceptually displayed.

3. Supplementary References

Galvagnion, C., Buell, A.K., Meisl, G., Michaels, T.C., Vendruscolo, M., Knowles, T.P., and Dobson, C.M. (2015). Lipid vesicles trigger alpha-synuclein aggregation by stimulating primary nucleation. *Nat. Chem. Biol.* 11**,** 229-234. 10.1038/nchembio.1750

Micsonai, A., Wien, F., Kernya, L., Lee, Y.H., Goto, Y., Refregiers, M., and Kardos, J. (2015). Accurate secondary structure prediction and fold recognition for circular dichroism spectroscopy. *Proc. Natl. Acad. Sci. U. S. A.* 112**,** E3095-3103. 10.1073/pnas.1500851112

Terakawa, M.S., Lee, Y.H., Kinoshita, M., Lin, Y., Sugiki, T., Fukui, N., Ikenoue, T., Kawata, Y., and Goto, Y. (2018). Membrane-induced initial structure of alpha-synuclein control its amyloidogenesis on model membranes. *Biochim. Biophys. Acta Biomembr.* 1860**,** 757-766. 10.1016/j.bbamem.2017.12.011
